# Towards a Personalized Vestibular Assessment in Older Patients with Cochlear Implant

**DOI:** 10.3390/jpm16020081

**Published:** 2026-02-01

**Authors:** Tiziana Di Cesare, Pasqualina Maria Picciotti, Walter Di Nardo, Daniela Rodolico, Jacopo Galli

**Affiliations:** 1 Audiology and ENT, Institute for Maternal and Child Health—IRCCS “Burlo Garofolo”, 34137 Trieste, Italy; tiziana.dicesare@burlo.trieste.it; 2 Department of Head, Neck and Sensory Organs, Università Cattolica del Sacro Cuore, 00168 Rome, Italy; walter.dinardo@policlinicogemelli.it (W.D.N.); jacopo.galli@policlinicogemelli.it (J.G.); 3 Unit of Otorhinolaryngology and Head-Neck Surgery, Fondazione Policlinico Universitario A. Gemelli IRCCS, 00168 Rome, Italy

**Keywords:** cochlear implant, vestibular function, older patients, elderly, personalized preoperative cochlear implant counseling

## Abstract

**Background**: Age-related vestibular decline frequently accompanies presbycusis, and older adults undergoing cochlear implantation (CI) may be particularly vulnerable to postoperative dizziness due to a reduced compensatory capacity and a higher burden of comorbidities. Although CI is an effective treatment for severe-to-profound sensorineural hearing loss in the elderly, its impact on vestibular function remains a critical concern. This study aimed to compare pre and postoperative vestibular performance in older patients (≥65 years) versus younger adults undergoing CI in order to identify the risk factors for postoperative vestibular deterioration and critical issues that characterize this category and carry out personalized preoperative counseling. **Methods**: In this monocentric observational study, adults undergoing CI were divided into two groups: older patients (OPS, ≥65 years) and younger patients (YPS, <65 years). Vestibular function was assessed preoperatively and one month postoperatively through a Dizziness Handicap Inventory (DHI), history of recurrent falls, clinical examination, video head impulse test (VHIT), bithermal caloric testing, and computerized dynamic posturography (Sensory Organization Test, SOT). Risk factors for postoperative vestibular worsening were analyzed using ANOVA test and chi-square statistics, with significance set at *p* < 0.05. **Results**: A total of 63 patients were included, with 18 surgeries involving OPS and 45 involving YPS. Preoperatively, OPS showed significantly higher rates of vestibular abnormalities on caloric testing (55.5% vs. 17.7% bilateral hyporeflexia, *p* < 0.05) and a higher prevalence of recurrent falls (33.3% vs. 4.4%, *p* < 0.05). Early postoperative dizziness (DHI^1^) increased significantly in both groups, but age ≥ 65 was a risk factor for ≥10% worsening (OR 2.2, *p* < 0.05). At one month, YPS returned to baseline DHI values, whereas OPS showed persistent dizziness with significantly higher DHI^2^ scores (29.2 vs. 12.9, *p* < 0.05). Vestibular worsening was identified in 33.3% of VHIT assessments and 44.4% of caloric tests in OPS, with caloric testing proving more sensitive than VHIT. Implantation on the better-functioning vestibular side and the presence of ≥3 comorbidities increased the likelihood of persistent postoperative dizziness. **Conclusions**: Older age is a significant risk factor for persistent dizziness and vestibular impairment one month after CI. Given the reduced compensatory capacity typical of older adults, vestibular assessment should play a central role in preoperative decision-making, particularly for side selection. Bithermal caloric stimulation is recommended as the most sensitive tool for detecting clinically relevant vestibular changes. Preoperative counseling for older CI candidates should include a detailed discussion of vestibular risks and the possible need for postoperative rehabilitation.

## 1. Introduction

Hearing rehabilitation in the elderly has undergone significant changes in recent years due to the ever-increasing spread of the problem and the reliability of new cochlear implant (CI) technologies. In fact, it has been estimated that by age 65, a third of people will have enough irreversible damage to hair cells in their inner ear to experience some degree of hearing loss (HL), causing nearly 226 million people over 65 worldwide to suffer from disabling HL who will require hearing rehabilitation [1,2]. This number is projected to double by 2050 considering the actual demographic transition towards an increasingly aging population [2].

The significant improvement of hearing performance in terms of speech recognition outcomes with CI among elderly patients with severe or profound HL, compared with the younger counterparts [3,4], has progressively raised the age of eligibility for surgery to the point of no longer considering it a limitation [5].

Several studies investigating the relationship between age and CI outcomes [5,6,7] highlight the importance of vestibular function in this population. Vestibular hypofunction is more frequently observed in CI candidates than in age-matched controls [7,8,9], and age-related factors may influence postoperative balance responses. This is particularly relevant given the close anatomical and physiological relationship between the auditory and vestibular systems, which are often affected by the same degenerative processes. Presbycusis—a progressive auditory impairment not attributable to otologic disease, ototoxic agents, or hereditary conditions—is associated with damage to hair cells, spiral ganglion neurons, and the stria vascularis [1,10]. In parallel, recent studies have focused on age-related vestibular changes and their impact on balance control [11,12]. Chronic dizziness affects almost 30% of individuals over 60 [13], prompting the Bárány Society to introduce the term presbyvestibulopathy to describe older patients with mild bilateral vestibular deficits and symptoms of imbalance or falls [14].

Age-related decline in multisensory integration, impaired visual function, polyneuropathy, central neurological conditions, and sarcopenia may further reduce postural stability and contribute to dizziness in the elderly [15]. Notably, dizziness is a common postoperative symptom—often influenced by anesthetic factors—particularly in older patients.

The aim of the present study was to evaluate the vestibular function before and after cochlear implantation in older patients, analyzing the impact of CI surgery on the vestibular system and the role of vestibular assessment compared to younger counterparts. Results could make clinicians aware of the risk factors for postoperative vestibular deterioration and critical issues that characterize this category in order to carry out personalized preoperative counseling.

## 2. Materials and Methods

It was a monocentric observational study approved by the Ethical Committee of Foundation Polyclinic University A. Gemelli IRCCS, Rome, Italy (protocol code 0012424/23; date of approval 13 April 2023). We included adult (>18 years) CI candidates affected by severe-to-profound SNHL. They were divided in two groups: the older patients (OPS) ≥ 65 years old and the younger patients (YPS) < 65 years old.

The surgical approach was conservative with the intracochlear placement of the array through the (extended) round window membrane except in the case of ossification, when the cochleostomy technique was performed. All patients had sufficient ability to read, understand, and complete the assigned questionnaires and sign an informed consent form. We included CIs of all brands.

Bilateral simultaneous implantations were excluded from the study; in case of bilateral sequential implantations, we considered only the first CI surgery. Bilateral sequential implantations with an interval time < 1 month between the two surgeries were also excluded. History of vestibular schwannoma, active middle ear disease, and congenital malformations of the auditory/vestibular system were exclusion criteria.

General history included a detailed interview about comorbidities, considering hypertension, ischemic heart disease, anxious–depressive syndromes, autoimmune disease, osteoporosis, thyroid disease, diabetes, epilepsy or other neurological disorders, oncological problems, and other diseases.

### Vestibular Evaluation

• The Dizziness Handicap Inventory (DHI) (Italian version by Nola et al. [16]) was administered 24–48 h before surgery (DHI^0^), 24–48 h after surgery (DHI^1^), and one month after surgery (before CI activation) (DHI^2^) to evaluate the pre, peri, and postoperative dizziness, respectively [9,16]. It consists of 25 multiple choice questions (“yes”—4 points, “sometimes”—2 points, and “no”—0 points), which provide a total score from 0 (no handicaps) to 100 (the greatest ailment imaginable). Values are considered normal (<10), borderlines (10–16), mild (18–34), moderate (36–52), and severe (≥54).
• Recurrent falls: Each patient was interviewed regarding the presence of recurrent falls in their daily life, providing a choice of 3 answers: “yes” (4 points), “sometimes” (2 points), and “no” (0 points).
• Vestibular assessment was performed 24–48 h before surgery and one month after surgery (before CI activation), including:
  - Clinical examination to assess the presence of spontaneous and/or positional nystagmus and to perform the head-shaking test (HST) and clinical head impulse test (HIT).
  - Video head impulse test (VHIT) using a VOG device (ICS Impulse, GN Otometrics, Taastrup, Denmark) to measure the gain of VOR (Vestibular–Oculomotor Reflex) for both the horizontal canals. It was performed with the patient sitting upright and fixating a visual target in front of him when the clinician generated head impulses by moving it abruptly and unpredictably in the horizontal plane. We considered normal VOR gain > 0.8 and normal gain asymmetry < 20% [9,17].
  - Bitermic caloric stimulation by the Fitzgerald–Hallpike technique (ICS Aircal Air Caloric Sprinkler Otometrics, Taastrup, Denmark). It was performed in a conventional manner (air-flow of 0.8 L/min at temperatures of 50 °C and 24 °C for 60 s, in the dark, in supine position with the head raised at 30°). Nystagmus amplitude was calculated by the system as slow phase velocity (SPV) and measured in °/s. The Jongkee’s formula was used to quantify the asymmetry between the sides. Results were expressed as unilateral weakness (UW normal < 15%) and directional preponderance degree (DP normal < 15%). Bilateral hyporeflexia was defined by the sum of the maximal peak velocities of the slow phase caloric-induced nystagmus for stimulation with warm and cold water on each side (SPV) < 25°/s [9,18].
  - Computed Dynamic Posturography (CDP) (Equitest, Neurocom Int. Inc., Clackamas, OR, USA) was performed with the patient standing on a dual footplate enclosed by a visual surround in six balance conditions (eyes open/closed; visual surround steady/rotated; platform steady/rotated—Sensory Organization Test (SOT)) as previously described [19]. For each test, we considered the Composite Equilibrium Score (CES) showing the weighted average of the different conditions and Sensory Analysis (SA) showing the contribution of the different sensorial afferences (somatosensory, visual, vestibular, and visual-preference). We considered normal CES and SA to be >70 [9,19].

## 3. Statistical Analysis

The sample was described in its clinical and demographic characteristics using descriptive statistics techniques. Continuous values with normal distribution, such as VOR gain and percentage of asymmetry on VHIT, were expressed as mean ± standard deviation (SD). Qualitative variables were expressed as absolute and relative percentages.

The ANOVA test was used to compare the mean scores of the DHI questionnaire, recurrent falls, VHIT, caloric test, and SOT before and after surgery in the two groups (older and younger patients).

The chi-square test (χ^2^ test and the odds ratios (OR) with 95% confidence intervals) was used to identify anamnestic and surgical risk factors for postoperative vestibular damage. For the purpose of the study, we considered as MCID (Minimal Clinically Important Difference) any value ≥ 10% (DHI, VOR gain on the implanted side, SPV nystagmus on the implanted side, and CES) after surgery compared to the starting score (for example, if a patient had a total preoperative DHI score of 50, any increment ≥ 5 points was considered significant). The choice to consider the MCDI as a percentage value and not as a fixed numerical value was dictated by the desire of the authors of the present manuscript to adapt the significance of the worsening score to the starting value of each patient [9]. The results were considered statistically significant for *p* < 0.05.

## 4. Results

We included 63 patients (36 F, 27 M; mean age 51.4 ± 15.6, range 18–84 years) with a complete vestibular assessment before and after.

Older patients (OPS) were 18 (10 F, 12 M; mean age 70.6 ± 15.9, range 65–84 years).

Causes of deafness were as follows: one genetic 5% (MELAS); one autoimmune cerebellar ataxia (5%); one viral infection 5% (one herpes zoster); one otosclerosis (5%); two drug ototoxicity (10.5%); three Meniere’s disease (15.8%); four Sudden Sensorineural Hearing Loss (25%); five presbycusis (25%).

They suffered from, on average, 2.5 comorbidities (range 0–6): 15 (83.3%) were affected by hypertension; 5 (27.7%) by ischemic heart disease; 3 by (16.6%) anxious–depressive syndromes; 4 (22.2%) by autoimmune disease; 1 (5.5%) by osteoporosis; 2 (11.1%) by thyroid disease; 7 (38.8%) by diabetes; 2 (11.1%) by epilepsy or other neurological diseases; 2 by (11.1%) oncological disease; 11 (61.1%) by other nonspecific diseases.

The younger group (YPS) included 45 patients (27 F, 18 M; men age 43.7 ± 15.8, range 18–64).

Their causes of deafness were genetic in 12 patients (26.6%) (9 related to connexin26 mutation and 3 syndromic, 1 Cogan, 1 Epstein, 1 Waardenburg); traumatic in 3 (6.6%) (1 electrocution and 2 temporal bone fractures); 1 (2.2%) meningitis; 4 (8.8%) otosclerosis; 1 (2.2%) chronic cholesteatomatous otitis media; 1 (2.2%) drug ototoxicity; 4 (8.8%) viral infections (1 cytomegalovirus; 1 paramyxovirus; 2 herpes zoster); 1 (2.2%) Sudden Sensorineural Hearing Loss; 18 (40%) idiopathic.

In regard to comorbidities, younger patients suffered from 0.95 disease, which was statistically lower than the elderly (*p* < 0.05). Seven (15.5%) were affected by hypertension; one (2%) by ischemic heart disease; six (13.3%) by anxious–depressive syndromes; three (6.6%) by autoimmune disease; three (6.6%) by osteoporosis; four (8.8%) by thyroid disease; four (8.8%) by diabetes; six (13.3%) by epilepsy or other neurological diseases; one (2.2%) by oncological disease; five (11.1%) by other nonspecific diseases.

The 100% (18/18) OPS vs. the 51% (23/45) YPS suffered from at least 1 disease (*p* < 0.05), the 39% (7/18) vs. the 22% (10/45) 2–3 comorbidities (*p* < 0.05), and the 39% (7/18) vs. the 4.5% (2/45) had >3 comorbidities (*p* < 0.05).

**Figure 1:**
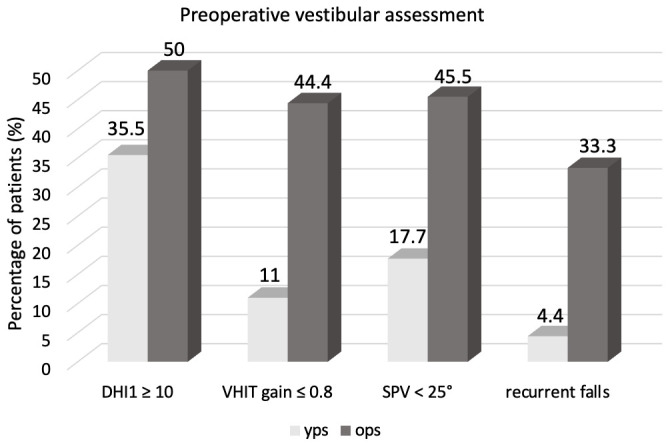
Preoperative vestibular assessment in younger (YPS) and older patients (OPS). DHI0: preoperative Dizziness Handicap Inventory; VHIT: video head impulse test; SPV: slow phase caloric-induced nystagmus velocity.

The preoperative vestibular evaluation showed no significant difference for the mean values of DHI between the younger and the older populations (13.4 vs. 19, *p* > 0.05). In any case, 16/45 (35.5%) younger patients reported abnormal values of DHI^0^ compared to the 50% (9/18) of the older patients (*p* < 0.05) (Figure 1).

A significant percentage of OPS (6/18, 33.3%) reported a history of recurrent falls compared to the younger counterparts (2/45, 4.4%) (*p* < 0.05) (Figure 1).

Five YPS (11%) had abnormal VOR gain (<0.8) in the side to be implanted; 8/45 (17.7%) had a significant asymmetry between the two sides (Figure 1).

Among the older population, 8/18 (44.4%) had an abnormal VOR gain in the side to be implanted (Figure 1); 7/18 (38.9%) had a bilateral hypofunction measured with the VHIT. Excluding three of them (one with autoimmune cerebellar ataxia and two with Meniere’s disease), 4/18 (22.2%) OPS were in accordance with the diagnostic criteria for the presbyvestibulopathy of the Bárány Society [14]. Five (27.7%) OPS had a significant asymmetry of the VOR gain.

The preoperative caloric stimulation showed an abnormal UW In 17/45 (37.7%) YPS compared to 7/18 (38.9%) OPS. Bilateral hyporeflexia (SPV < 25%) resulted in 8/45 (17.7%) YPS and 10/18 OPS (55.5%) (*p* < 0.05) (Figure 1). Consequently, 7/18 (38.9%) older patients met the criteria for the presbyvestibulopathy using the caloric stimulation.

The mean preoperative Composite Equilibrium Score (CES) of the Sensory Organization Test (SOT) was 54 in OPS (85 somatosensory, 35.3 vestibular, 72.5 visual, and 72 visual-preference) and 71 in YPS (92.6 somatosensory, 54.6 vestibular, 72 visual, 87 visual-preference) (*p* < 0.05 for vestibular scores comparison).

**Figure 2:**
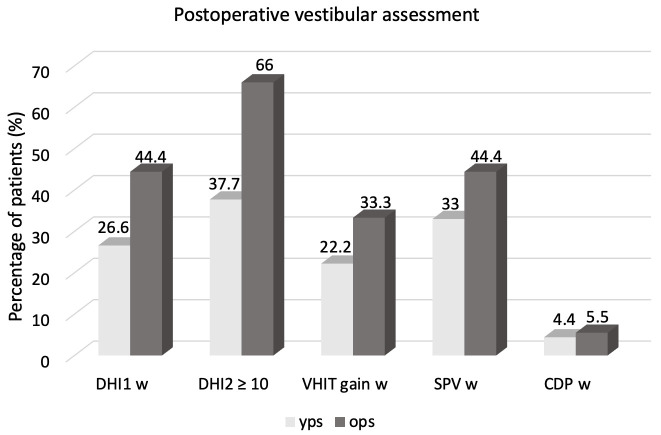
Postoperative vestibular assessment in younger (YPS) and older patients (OPS). DHI1: Dizziness Handicap Inventory 24–48 h; VHIT: video head impulse test; SPV: slow phase caloric-induced nystagmus velocity; w: significant worsening; DHI2: Dizziness Handicap Inventory 1 month.

The early (24–48 h) postoperative DHI^1^ was 23.2 for YPS and 29 for OPS, without a significant difference (*p* > 0.05). In any case, in both groups the early postoperative increase in dizziness perception was significant (*p* < 0.05). The age ≥ 65 represented a risk factor for a significant (≥10%) worsening of the DHI 24–48 h after surgery (OR = 2.2; CI = 0.7–6.8; *p* < 0.05) (Figure 2). In both groups, the presence of ≥3 comorbidities was a risk factor (OR = 2.05; CI = 0.5–7.7; *p* < 0.05).

The late postoperative evaluation showed a significant difference between the two groups for the mean DHI^2^ (12.9 vs. 29.2, *p* < 0.05). Consequently, while most young patients showed a decrease in the total score compared to the immediate postoperative period, with no difference between DHI^0^ and DHI^2^ in this group (*p* < 0.05), most older patients demonstrated a persistence of the dizziness perception 1 month after surgery, with a significant increase compared to the preoperative evaluation (*p* > 0.05). The age ≥ 65 years was a risk factor for the presence of dizziness after 1 month (OR = 3.3; CI = 1–10.4; *p* < 0.05) (Figure 2).

In 16/63 cochlear implantations (25.4%), the VHIT showed a significant worsening (≥ 0.1) of the VOR gain in the implanted side after surgery; it involved 6/18 (33.3%) surgeries performed in OPS and 10/45 (22.2%) surgeries performed in the younger population (OR = 1.75; CI = 0.5–5.8; *p* < 0.05) (Figure 2).

The chronic bilateral hypofunction or the diagnosis of presbyvestibulopathy were not related to the worsening after surgery.

In 23/63 ears (36.5%) the caloric test demonstrated a significant worsening of the SPV in the implanted ear after surgery, involving 8/18 (44.4%) surgeries performed in OPS and 15/45 (33%) in YPS (OR = 1.6; CI = 0.5–4.9; *p* < 0.05) (Figure 2). In both groups, the caloric test was more sensible compared to VHIT.

As for VHIT, the preoperative bilateral vestibular hyporeflexia or the diagnosis of presbyvestibulopathy were not risk factors for a significant reduction in the evoked nystagmus with the caloric test. Among the elderly, Meniere’s disease (OR = 4.8; CI = 0.35–65.7; *p* < 0.05) and the presence of ≥3 comorbidities (OR = 2.2; CI = 0.2–20.4; *p* < 0.05) were risk factors for vestibular damage.

In both groups, the cochlear implantation in the hyper-functioning side, in case of asymmetry, was related to the persistence of postoperative dizziness after 1 month (*p* < 0.05).

In both groups, the mean postoperative scores of CDP were not different (*p* > 0.05). Only three patients in the total sample (4.7%) demonstrated a significant worsening of the CES, 1/18 (5.5%) in OPS and 2/45 (4.4%) in YPS (*p* > 0.05).

## 5. Discussion

This study provides a comprehensive evaluation of vestibular function before and after cochlear implantation in adults over 65 years of age, comparing outcomes with a younger cohort. The findings confirm that advanced age is associated with both a higher prevalence of preoperative vestibular dysfunction and a greater likelihood of persistent postoperative dizziness.

Consistent with the previous literature reports, the older cohort in our study presented a significantly higher proportion of vestibular deficits before surgery—both in VHIT and caloric testing—than younger participants. This aligns with current pathophysiological models of presbyvestibulopathy, which describe age-related degeneration affecting hair cells, vestibular nerve fibers, and central pathways involved in balance control, as well as age-related cochlear degeneration (presbycusis) [10,11,12,13,14]. Notably, 44–55% of older patients met diagnostic criteria for presbyvestibulopathy, which is comparable to prevalence estimates reported by the Bárány Society in individuals over 70 years of age [14]. Likewise, the markedly higher incidence of recurrent falls among older adults correlates with prior findings showing that nearly one-third of people over 60 experience chronic dizziness or imbalance, with substantial repercussions for daily function and fall risk [12,13,20]. Lovin et al. [7] have also shown that older cochlear implant candidates are more likely than age-matched controls to present vestibular hypofunction, supporting the hypothesis of shared vulnerability between auditory and vestibular structures. The significantly higher burden of comorbidities in the older group—many of which interfere with multisensory integration and motor control—further contributes to this predisposition.

Regarding our vestibular findings after CI, both age groups reported a significant increase in dizziness during the first 24–48 h after surgery, in agreement with the literature [8,9]. However, age ≥ 65 years emerged as a clear predictor of greater early postoperative worsening on the Dizziness Handicap Inventory, reflecting a reduced short-term vestibular compensatory capacity in older adults. Indeed, previous research suggests that aging is associated with reduced cerebellar plasticity, slower sensory rebalancing, and reduced postural reserve, all of which may delay early compensatory mechanisms [11,12]. Furthermore, caloric stimulation has been shown to be more sensitive than VHIT in detecting early postoperative vestibular changes, a finding consistent with recent meta-analyses and clinical studies [8,17,18]. This increased sensitivity of caloric testing is particularly relevant in older adults, who often exhibit early deficits in low-frequency vestibular responses, which caloric testing is better suited to detect [12].

On the other hand, the one-month evaluation after CI highlights a highly significant finding regarding the persistence of dizziness in the older cohort, in contrast to the recovery observed in younger patients. Age ≥ 65 years was a significant independent risk factor for persistent symptoms at one month. We hypothesize that the high prevalence of bilateral vestibular involvement before implantation and comorbidities, including polyneuropathy, visual impairment, metabolic and neurological disorders, and sarcopenia, hinder vestibular compensation. Reduced efficacy of vestibular rehabilitation in older adults has also been described in the literature, supported by neurophysiological evidence [12]. In our study, the presence of more than three comorbidities was associated with early and persistent postoperative dizziness, highlighting the importance of a multidimensional geriatric assessment both before and after CI.

Another important finding of our analysis concerns the side to be implanted. Our data confirm that implantation on the side with better function in cases of preoperative vestibular asymmetry is associated with a higher probability of persistent dizziness, in agreement with previous studies [7,9]. This underlines the clinical relevance of preoperative vestibular testing to guide side selection, particularly in older adults who show slower and less complete compensation.

Based on our results, several clinical implications emerge. The first concerns the need for a comprehensive vestibular evaluation in CI candidates, especially those ≥65 years of age, both to identify presbyvestibulopathy and to guide surgical planning. This evaluation should include the bithermal caloric test, which has demonstrated greater sensitivity than VHIT. The second consideration concerns the general evaluation for possible comorbidities, given their significant contribution to postoperative disequilibrium. The third point concerns the need for preoperative counseling to explicitly address the increased risk of persistent dizziness in older adults, even when a favorable hearing outcome is expected. Patients should also be informed of the possibility of vestibular rehabilitation, which should be strongly recommended to reduce the risk of falls and facilitate recovery.

Finally, we must necessarily state some limitations of the present study, which concern the small number of the sample examined, the absence of an assessment of vestibular potentials (VEMPs), and the lack of a long-term follow-up that would provide a clearer understanding of the temporal course of vestibular compensation.

A further limitation is the lack of assessment of vestibular-evoked myogenic potentials (VEMPs). It is known that the otolithic organs, particularly the saccule, are vulnerable to both age-related degeneration and cochlear implant-related trauma, and isolated otolithic dysfunction can occur even in the presence of preserved semicircular canal function [21,22,23]. Therefore, vestibular assessment using VHIT and caloric testing may underestimate the extent of vestibular damage in elderly patients. This limitation may be clinically relevant, as otolithic dysfunction can be associated with impaired postural control and an increased risk of falls, especially in the elderly [21,24]. Therefore, the lack of VEMP assessment in our study may have led to an underestimation of fall risk and may partly explain the persistence of dizziness and imbalance observed in elderly patients despite normal canal function.

Future studies should, therefore, include a comprehensive vestibular assessment incorporating otolithic function tests to better characterize vestibular impairment and improve fall risk stratification in elderly cochlear implant candidates.

## 6. Conclusions

An age over 65 years is a risk factor for the persistence of vertigo symptoms one month after cochlear implant surgery. Considering the potential difficulty of performing vestibular rehabilitation in elderly patients in the postoperative period, the hypofunctioning side should be chosen whenever possible. The preferable test is bithermal caloric stimulation as it is more sensitive than VHIT. Aspects relating to vestibular function of the elderly patient should be given attention in the preoperative stage in order to personalize counseling and postoperative treatment.

## Data Availability

The raw data supporting the conclusions of this article will be made available by the authors on request.
